# Emotional Labor and the Problem of Exploitation in Roboticized Care Practices: Enriching the Framework of Care Centred Value Sensitive Design

**DOI:** 10.1007/s11948-024-00511-2

**Published:** 2024-09-11

**Authors:** Belén Liedo, Janna Van Grunsven, Lavinia Marin

**Affiliations:** 1 Institute of Philosophy, Spanish National Research Council, Madrid, Spain; 2 Ethics and Philosophy of Technology Section, TU Delft, Delft, The Netherlands

**Keywords:** Assistive robotics, Care-centred value-sensitive design, Emotional labor, Ethics of care, Paid care work, Roboethics

## Abstract

Care ethics has been advanced as a suitable framework for evaluating the ethical significance of assistive robotics. One of the most prominent care ethical contributions to the ethical assessment of assistive robots comes through the work of Aimee Van Wynsberghe, who has developed the Care-Centred Value-Sensitive Design framework (CCVSD) in order to incorporate care values into the design of assistive robots. Building upon the care ethics work of Joan Tronto, CCVSD has been able to highlight a number of ways in which care practices can undergo significant ethical transformations upon the introduction of assistive robots. In this paper, we too build upon the work of Tronto in an effort to enrich the CCVSD framework. Combining insights from Tronto’s work with the sociological concept of *emotional labor*, we argue that CCVSD remains underdeveloped with respect to the impact robots may have on the emotional labor required by paid care workers. Emotional labor consists of the managing of emotions and of emotional bonding, both of which signify a demanding yet potentially fulfilling dimension of paid care work. Because of the conditions in which care labor is performed nowadays, emotional labor is also susceptible to exploitation. While CCVSD can acknowledge some manifestations of unrecognized emotional labor in care delivery, it remains limited in capturing the structural conditions that fuel this vulnerability to exploitation. We propose that the idea of *privileged irresponsibility,* coined by Tronto, helps to understand how the exploitation of emotional labor can be prone to happen in roboticized care practices.

## Introduction

Healthcare sectors across the globe are investing in adopting assistive robots, that is, robots intended to perform in care settings. Confronted with an influx in (aging) patients and increasing staff shortages, the hope is that robots can help provide more efficient care by assisting with (or even replacing) some of the care tasks traditionally performed by humans (Kyrarini et al., 2021). Much like other technology-driven transitions in healthcare, this ‘robotic transition’ harbors ethical and political implications that require ethical analysis. The ethics of care has provided fruitful resources for taking up this task (Vallor, 2011, Van Wynsberghe, 2015, Vandemeulebroucke & Gastmans, 2020, Stokes & Palmer, 2020, Pirni et al., 2021, Yew, 2021, Li, 2022). One of the most prominent —if not *the* most prominent— care ethical frameworks for assessing the ‘robotic transition’ in healthcare contexts has been developed by Aimee Van Wynsberghe: the Care-Centred Value-Sensitive Design or CCVSD^1^ (Van Wynsberghe, 2013, 2015). Van Wynsberghe has adapted the work of care ethicist Joan Tronto to the case of assistive robotics, proposing a model of Value Sensitive Design in coherence with the values of care. Her normative proposal mainly focuses on healthcare delivery by foregrounding the dynamics and qualities of interpersonal interactions in care settings. Van Wynsberghe’s work has undoubtedly helped surface a number of ethically pertinent considerations with respect to assistive robots. However, as we argue, an important and highly relevant layer of analysis of Tronto’s ethics of care is still missing.

The need for such an analysis emerges when attending to the specific setting within which assistive robots are expected to be predominantly implemented, namely institutional settings in which care is performed for a wage. For reasons we discuss below, *paid* care work is a form of work particularly susceptible to exploitation. We argue that a care ethical approach to assistive robotics should also attend to their impact on the labor conditions of paid care workers (PCWs). Those labor conditions are, to an important degree, marked by the performance of *emotional labor*. That is, in labor contexts, PCWs are expected to take responsibility for the emotions of people in their care as a constitutive dimension of their labor duties. We build upon the care ethics proposal by Tronto to point out the conditions that make emotional labor prone to exploitation and the ways robots can contribute to maintaining such potentially exploitative conditions. We contend that, thus far, the scope of CCVSD does not sufficiently address this problem, even though it is a significant part of Tronto’s work, and that this gives rise to a significant blind spot in how we evaluate the impact of robots on care workplaces.

The structure of this paper is as follows. First, we outline the main contributions of Van Wynsberghe’s CCVSD and the gaps that may be found in it regarding emotions in care and labor conditions of PCWs. Then, we introduce the concept of emotional labor as a tool for addressing this gap. We subsequently turn to the work of Tronto to explain the structural mechanisms that make emotional labor vulnerable to exploitation. In particular, we will expound the idea of *privileged irresponsibility* and attend to its possible manifestations in care practices. Based on this conceptualization, we point out the insufficiency of CCVSD as an exclusively design-focused approach for addressing the structural conditions that shape care practices and, thus, its limitation for fully recognizing care values in roboticized care.

## Care Ethics and Assistive Robots: the Care-Centered Value-Sensitive Design

Van Wynsberghe’s CCVSD (Van Wynsberghe, 2013, 2015, 2016) arguably offers the most developed endeavor to apply the tradition of ethics of care to the ethical assessment of assistive robotics. CCVSD highlights the centrality of concepts such as intimacy, trust, responsibility, and vulnerability in assessing how robots, when introduced in existing care settings and practices, might transform those settings and practices. Care is understood as a practice which is developed through time, shaped by the relationships between the people involved, and driven by a moral commitment to the wellbeing of the other (Van Wynsberghe, 2015). In Van Wynsberghe’s words, a care practice refers to:

The attitudes, actions and interactions between actors (human and non-human) in a care context that work together in a way that manifests care values: a care practice facilitates the realization of care values. (Van Wynsberghe, 2015, p. 27)

Van Wynsberghe builds upon the work of Joan Tronto and her scheme of care (Tronto, 1993). Care, according to the first version of Tronto’s proposal, comprises four interconnected, non-linear stages (caring about, caring for, care-giving, and care-receiving), each of them guided by a particular moral value (attentiveness, responsibility, competence, and responsiveness). CCVSD combines this scheme with the Value-Sensitive Design framework (Friedman et al., 2001), which understands design as value-laden and proactively aims to embed ethical values in R&D processes. On this basis, the objective of CCVSD is to support the development of assistive robots that are capable of respecting and promoting the values of care.

Importantly, Joan Tronto proposed adding a fifth phase of care in her 2013 book *Caring Democracy*: *caring with* (Tronto, 2013). This phase is shaped by the values of trust and solidarity, and it refers to citizens’ shared responsibility for allocating care duties in a fair manner. This new phase is aligned with Tronto’s fundamental commitment to a politization of ethics of care capable of grasping the relevance of justice and equality in care allocation at a social level. Van Wynsberghe’s adaptation does not include this phase, even if, as we will develop in the next paragraph, her attention to context is relatively akin to Tronto’s attention to social organization of care duties. In our view, the absence of this fifth phase can obscure the possibility of addressing the structural conditions that shape care practice’s ethical relevance. While a comprehensive account on the ways in which robot development can align with the value of “caring with” is not the main purpose of this article, our argument can be seen as a contribution to develop Tronto’s political framework for the analysis of robotiziced care.^2^

Van Wynsberghe explicitly highlights *context* as one of the elements that should be thoroughly scrutinized when evaluating the ethical desirability of an assistive robot:

Firstly, one must identify the context within which the care practice is taking place. On the one hand, the context determines the structure of care, the resources available, and the various routines in place for patients and personnel. In this sense, structural context refers to the specific hospital and ward vs. a nursing home vs. a home setting. On the other hand, context can refer to a cultural climate that plays a role in how things are seen and done. (Van Wynsberghe, 2015, pp. 70–71)

In attending to context, CCVSD mainly addresses formal healthcare contexts, since the current development of assistive robotics is largely centered on collaborating with nurses and physicians in their daily tasks. As a result, CCVSD seems already attuned to institutional contexts where care is performed for a wage. However, while we endorse Van Wynsberghe’s focus on context, and we agree that the “cultural climate” may play a key role in “how things are seen and done” in the shaping of care practices, we believe that CCVSD does not sufficiently attend to some of the distinctive features of care being performed as a job in much of these contexts.^3^

A similar case can be made for the attention paid to the implications of emotionality for caregivers within CCVSD. CCVSD provides tools for addressing the capacity of PCWs to suitably engage in emotional relationships when a robot enters the scene. For instance, Van Wynsberghe displays the example of a lifting robot that may help nurses in the physically exigent task of lifting patients, but that may also disrupt opportunities for developing a close relationship with the patient. In this sense, CCVSD grasps the importance of emotionally charged relationships between nurse practitioner and patient, forged through time and interpersonal engagement. From CCVSD, we see that lifting qua practice is not only an activity aimed at moving a person from point A to point B. It is also a moment in which the nurse may attend to the emotional status of the patient and in which they can create a bond. Introducing a robot in this scenario should be done with an awareness of all these emotionally charged aspects of care practices.

Hence, in one sense, Van Wynsberghe certainly addresses the relevance of emotions in care and the way in which engaging in and managing emotions is part and parcel of meaningful care work. Furthermore, she focuses on the limitations robots show in engaging with humans in this emotionally charged respect. While roboticists are attempting to incorporate new capacities to overcome these limitations —capacities such as emotion recognition and emotionlike performance— Van Wynsberghe persuasively concludes that assistive robots are far from capable of responsibly engaging in the aspects of care that require emotional efforts. CCVSD thereby encourages an evaluation of assistive robots that focuses on whether and how robots might affect the ways in which a nurse takes care of the emotional wellbeing of the patient.

We argue that the implications of the emotional aspects of care for the paid care worker are not sufficiently addressed in this account. When emotional efforts happen as part of professional duties, they acquire the category of emotional labor. As we will show, this emotional labor can be subjected to exploitation, marked by gender and race discrimination patterns visible at a global level. When robots are incorporated in the care-and-work spaces, they can modify the responsibilities and burdens of workers in ways that can reinforce or (re)introduce such exploitation. Here, we want to address the specific impact robots can have on the emotional labor characteristic of care jobs. To do so, we will now delve into the concept of emotional labor and show how it can help to point out the structural vulnerability to exploitation in contexts of paid care work that is insufficiently attended to in CCVSD.

## Emotional Labor: What Counts as Good Care for a Care Worker?

Care jobs are constitutively emotion-laden. Among other responsibilities, caregivers should also acknowledge the emotions of people under their care and adequately respond to them. This, in turn, requires that care workers are capable of coping with their own potentially triggered emotions. That is, PCWs must take charge of the emotional reality of personal interaction in order to perform their job correctly. Importantly, this professional duty of emotional engagement is ambivalent, since it can be a source of satisfaction but also of personal struggle and exploitation. The concept of emotional labor helps to understand the nuances of this situation.

*Emotional labor* is a term coined by Arlie Hochschild, (1983) that describes the kind of emotional performance required from service workers in order to make the customer feel a certain way. Hochschild recognizes that emotional labor in turn requires *emotional management*, that is, the conscious handling of emotions, which responds to social norms that state the acceptability of different emotional displays in different contexts. This emotional management (or regulation) is not itself harmful; actually, it is a common part of people’s experience of emotion and is required for a successful interpersonal exchange. However, Hochschild argues that service jobs require an excessive acting out of emotions from workers that does not align with their inner state, which may be a form of labor exploitation. Performing emotions in exchange for a wage differs from performing them in spontaneous, quotidian social relationships, in the sense that the requisites may be specifically settled by employers. Because of that, there is a potential distance between the inner sense of self and purpose of employees and the type of emotional display required.

Originally, Hochschild mainly analyzed the case of flight attendants, theorizing about a new form of abusive conditions imposed on workers in capitalist markets: workers not only sell their material workforce but also their emotional capacities and intimate feelings. Although care jobs were not the main concern in Hochschild’s seminal account, they have been highlighted in the subsequent literature, given the fundamental role played by emotions in care contexts (Bolton, 2001; Elliot, 2017; Himmelweit, 1999; James, 1992; Mann, 2005; Rodriguez, 2014; Stacey, 2011; Ward & McMurray, 2015). The main difference between the emotional labor required in a service job and the one required in a care job is that, in the latter, the managing of emotions is not only a market-oriented feature but a fundamental trait of good care. Furthermore, when care jobs are vocational, the alignment between an inner sense of self and the requirements of the job is more likely to happen. Various empirical investigations suggest that, contrary to flight attendants, female PCWs can adapt relatively smoothly to the exigencies of emotional labor, with the emotional bonding with clients and patients usually counting as a source of satisfaction and personal flourishing (Bolton, 2001; Duffy et al., 2015; Franzosa et al., 2018; Stacey, 2011; Wright, 2023).

Exploitative labor happens when someone benefits from the work of someone to the detriment of her interests (Brake, 2020).^4^ In the case of PCWs, to whom we limit our analysis, the benefit may come in the form of economic profit, avoidance of the fair share of work, or social credit. The harm to the best interests of PCWs manifests in substandard labor conditions (International Labour Organization, 2013) and unequal distribution of duties and profits of care (Brake, 2020), and it is fueled by gender and race stereotypes (Cameron & Moss, 2007; Duffy, 2011). In this sense, while we do not commit ourselves to any strong claim regarding the existence of a gender-based exploitation in care system as a whole, we do argue that there is a specific type of labor exploitation in care labor that is affected by gendered and racialized axes of oppression.

Accordingly, it can be stated that the role of emotional labor in care workers’ experience takes the form of a paradox that can make it difficult to notice exploitation. The personal identification of (many) women with their caring roles and their emotional requisites is a major source of meaning and flourishing in their work lives. However, this very identification and satisfaction may function as part of an exploitation dynamics. Jan Slaby (2016) has investigated how personal satisfaction intertwines with adherence to existing labor structures and even self-exploitation. In this way, it is possible that PCWs’ personal gratification with the emotional nature of their work contributes to them assuming deficient work conditions, and thus potential exploitation can remain unacknowledged by them or by other agents. Furthermore, expectations about female workers’ personal commitments to caring can limit the benefits offered in this kind of jobs, since they are deemed to be performed primarily ‘out of the heart’.

In what follows, we will delve further into this paradox of emotional labor: first, we will consider the experience of care workers and the importance they attribute to emotions in performing and enjoying their work. After that, we will turn to Tronto’s concept of privileged irresponsibility for arguing that exploitation practices are shaped by the structural conditions of care duties’ allocation, that such structural conditions are insufficiently attended to in CCVSD, and that those wider structural factors should be included in a thorough analysis of the impact of assistive robots on PCWs.

### Workers’ Experiences of Emotional Labor

Following several empirical investigations, PCWs usually strive to achieve a balance between stressful working conditions and personal satisfaction with their work (Aronson & Neysmith, 1996; Himmelweit, 1999; Rodriguez, 2014; Stacey, 2011). This satisfaction is linked to their personal identification with the caring nature of their profession: they commonly feel that care work is more than just a job. Some of the testimonies in these studies sharply illustrate this motivation:

I get great satisfaction out of what I do. That’s the only reason I’m still in it. Because there’s no money in it. There are no benefits to it. But without me—this is going to sound really conceited—but without me or people like me, most of them cannot stay at home. (Stacey, 2011,  p. 114)

So many times they treat us like part of the family. It’s not just work. (Aronson & Neysmith, 1996, 66)

Aides, namely, personal health assistants, are one of the less privileged groups in the care landscape. Emotional labor is highly relevant in both their duty and their identity (Stacey, 2011). In one sense, this emotional labor does not seem to be intrinsically harmful to them. They usually proudly engage in close relationships with their clients, caring genuinely about their well-being. For example, a young aide employed at a nursing home reports that:

I like to be with older people. When I saw them I saw my mom or my dad. I always open my heart for them. Even though it’s so hard, but I don’t care because I like to work. (Berdes & Eckert, 2007, p. 344)

Similarly, studies show that nurses, much like aides, tend to find their personal relationships with patients to be the most rewarding element of their job (Utriainen & Kyngäs, 2009), and that they identify closely with caring. For example, a nurse stated that: “the essential basis of nursing is caring. You can't be a nurse if you don't care” (Bolton, 2000, p. 583). Another study analyzing nursing students’ experiences concluded that recognition of emotional labor is critical to the proper training of students, and collected similar testimonies:

But then I have learnt how to have sympathy, not only sympathy and even empathy. Because when we have a patient right in the ward, there is a certain connection between me as a nurse, and the patient regardless of their status. (Msiska et al., 2014, p. 47)

Conversely, as various studies indicate, the main problems that different PCWs outline concern factors that precisely undermine the possibility of providing good care through emotional labor. One of them is the lack of support from their employers, for example, when they face the death of a client (Franzosa et al., 2018). Some other problems include conflicts with families (Delp et al., 2010); contentious patients who refuse to engage in honest relationships with them and who commodify them, including being treated “as a maid” (Franzosa et al., 2019; Ming et al., 2023; Salazar Parreñas, 2015); and a lack of peer support due to the absence of common spaces and complicated schedules (Bourgeault, 2015).

Some have proposed to understand these problems as related to a deficit of recognition of their emotional labor (Franzosa et al., 2019; Ming et al., 2023) while others maintaining that such recognition would significantly improve nurses’ work conditions (Mann, 2005). It has also been suggested that the lack of recognition and support for emotional labor is a major reason for nurses to abandon their jobs (Elliot, 2017). We align ourselves with these views, maintaining that PCWs would benefit from a much higher recognition^5^ for their emotional labor.

We suggest, then, that exploitation of emotional labor happens in at least two forms. In one sense, when PCWs are alienated from their sense of purpose (e.g., chaotic shifts that do not allow them to forge authentic relationships with clients), they find themselves in a place more alike to Hochschild’s flight attendants, with emotional labor being a mere service being sold to their employers in an alienating way. In a second sense, even if PCWs maintain a certain authenticity and thus avoid this type of personal alienation, institutions and employers may still harm their interests by maintaining their subordinate position while profiting from the gains of their efforts. In this second sense, the personal satisfaction with the requisites of caring jobs could function precisely as a way of masking the exploitation.

To explain this second form in more depth, we will now argue that PCWs are vulnerable to exploitation partly because care duties are unequally distributed and affected by gender, class, and racial prejudices. For doing so, we turn to a discussion of the concept of *privileged irresponsibility*, developed by Tronto, which will help to grasp a critical point of attention within roboticized care that is currently underappreciated in CCVSD.

### The Conditions of Exploitative Emotional Labor: Back to Joan Tronto

Why is emotional labor rarely recognized adequately through structural mechanisms, despite its widely acknowledged fundamental role in caring? Joan Tronto’s concept of privileged irresponsibility, introduced in *Moral Boundaries* (1993) and further developed in *Caring Democracy* (2013), can help to shed light on the social dynamics that make such a situation possible and how to tackle its persistence in roboticized care work.

Briefly put, Tronto argues that care responsibilities are differently allocated among people in liberal-democratic capitalist societies because care has been mistakenly designed as an exclusively moral issue, excluded from the political discussion, as well as labeled as a feminine and private task (Tronto, 1993). As a consequence, care responsibilities are nowadays distributed on the basis of veiled assumptions about the presumed duty of some people to care and the alleged right of some other people not to care. This alleged right is based on the claim that the latter have already contributed to the ‘care’ of people in some other way (e.g., bringing a salary home). This form of eluding responsibilities for care is what Joan Tronto names “privileged irresponsibility”, and it is the consequence of the belief that care is a feminized and private task. To this day, care tends to be assigned either to the nuclear traditional family, with its gendered distribution of responsibilities, or, increasingly, to the market, in which the axis of gender, class, and race also implies that some people are expected to care more than others. Against that, Tronto argues that democratic care allocation should include the effective participation of everyone in the decisions about the allocation of care responsibilities, as well as a critical examination of the privilege of not caring currently enjoyed by some groups of people (Tronto, 2013). Among the responsibilities that are unfairly distributed, emotional labor stands out as unrecognized and particularly relevant.

Privileged irresponsibility is a structural, global tendency that helps to explain why caring tasks are unequally distributed among groups. As highlighted by subsequent research, privileged irresponsibility contributes to maintain the inequality between groups by reinforcing the prestige status of those who are benefited by it (Bozalek & Zembylas, 2023). Tronto proposes that privileged irresponsibility works both at the moral level and at the political level (Tronto, 2013, p. 58). Morally, it is a mechanism that allows some people to avoid caring in interpersonal contexts, such as households or hospital rooms, by claiming that caring is not their business. For example, Tronto analyzes how the breadwinner family model characteristic of middle-class US families in the mid-twentieth century allowed the man to exempt himself from caring by contributing to the economic sustaining of the family. Politically, it is a form of power held by some groups of people that enables them to put too many responsibilities on the shoulders of those who are supposed to perform care. For example, migrant domestic workers are increasingly taking over caring tasks all over the Global North as a form of cheap, undervalued labor that usually lacks proper legal protection (Salazar Parreñas & Silvey, 2018). The expectations about who is supposed to care are historically shaped by gender, class, and race stereotypes (Duffy, 2011), and allows privileged groups to rely on the caring labor of disadvantaged groups. Emotional labor is also part of this unequal distribution of responsibilities. The fact that some people continue to be able to free themselves from care duties just by exerting their privileged irresponsibility makes PCWs vulnerable to this specific form of exploitation.

An ethical assessment of the role of emotional labor in roboticized care workspaces requires a two-step analysis. Firstly, the ethical appraisal should make explicit the existence of emotional labor, and the recognitive mechanisms needed by PCWs in order for emotional labor to be a source of satisfaction. At the level of design, this could mean attending to whether a robot might exacerbate the challenging exercise of emotional labor, or, ideally, whether it can work as an ally for PCWs. This is important both for the success of care itself and for avoiding harming PCWs because of stress and overburdening. At the level of implementation, this could mean attending to the training needed to handle a new robot and the ways in which this training might disrupt forms and moments of recognition that make emotional labor manageable if not enjoyable.

Secondly, as we have proposed in this section, we should keep in mind that one of the reasons why emotional labor is likely to be subjected to exploitation is an implicit acceptance of privileged irresponsibility. How can this notion support assessments of a good care work environment and the role robotics may play in this environment? In the next section, we argue that an exclusively design-focused approach such as CCVSD is insufficient for addressing the structural determinants of the exploitation of emotional labor.

## Rethinking Care-Centered Value Sensitive Design Through the *Lens* of Emotional Labor

In this section, we argue that CCVSD falls short of a comprehensive understanding of the particularities of emotional labor, specifically its vulnerability to exploitation. As a consequence, its overall capacity for upholding care values in roboticized care is limited. In fact, we worry that, without attending to the way in which privileged irresponsibility might operate in those contexts, the goal of promoting good care via design interventions might be self-undermining by contributing to the continuation of privileged irresponsibility. In what follows, we outline how the notions of emotional labor and privileged irresponsibility help to understand the exploitation problems that can continue to be enabled by assistive robots, even when they are designed in ways that recognize some aspects of good care delivery.

As mentioned, Van Wynsberghe (2015) indicates that the context of a specific care practice should be considered when evaluating an assistive robot. The responsibility allocations within a care practice should be explained in detail so as to assess how the robot might impact the workload of every person involved and the workflow within the team. While this evaluation can contribute to a smooth incorporation of the robot in the care practice, it does not sufficiently acknowledge the relevance of the structural political factors that frame the sharing of responsibilities within the care practice. The existence of privileged irresponsibility creates conditions under which some people tend to disproportionately carry the responsibilities of emotionally attending to care-receivers. Given that robots are unlikely to be capable of performing successful emotional labor any time soon (if ever), and given that they will likely transform the sharing of activities and responsibilities within the human workforce, it is essential to be aware of the ways in which exploitation of emotional labor is prone to happen in the robotized care workplace if not tackled. Below, we offer a few examples that illustrate our worry.

In overburdened healthcare systems, robots are often presented as solutions to problems of inefficiency (Maibaum et al., 2022). It is a genuine concern whether this is indeed the case. Evidence of the impact of robots in the care workforce is still limited, but research points out that poor embedding of new technologies and inattention to proper training can actually make robot-involving care work less efficient (Hamblin, 2022; Wright, 2023).

Even if robots are able to live up to their efficiency hype, it is a further question whether this is desirable from a care perspective. Robots might optimize an unfair structure marked by privileged irresponsibility, thus contributing to its persistence and masking the exploitative conditions we have presented here. To unpack this concern further, we turn to a case presented by Van Wynsberghe herself, which she presents as an example of a possible prospective development of an assistive robot compliant with CCVSD. We, however, point out the importance of analyzing this case further through the lens of privileged irresponsibility.

The example concerns the hypothetical *wee-bot*, which Van Wynsberghe imagined for tackling a problem noticed in some hospitals, where nurses do not have sufficient time and resources to collect urine samples from oncological patients safely. Nevertheless, they still engage in the task of urine collection, risking their own safety in the process (since the urine contains toxins whose manipulation is dangerous). This is a situation where the unsatisfactory conditions of performing care are resolved by exploiting the commitment of PCWs to the value of care, resulting in the prioritization of care for the patient over their own well-being and safety. The *wee-bot* would be designed to appropriately acknowledge the care values that should be respected in this specific care practice, enabling it to perform the task of urine sample collection in a way that is dignifying for the patient, safe for everyone in the institution, and that helps to liberate PCWs from an exploitative dimension of their current situation. Van Wynsberghe adds that even if the *wee-bot* is put in place, the role of the nurse will still be important for accomplishing the whole objectives of care, particularly the emotional aspects of the relationship.

In this example, the *wee-bot* responds to the immediate needs of those who give and receive care. The *wee-bot* does seem to be contributing positively to one way in which emotional labor can be exploited in contexts of unsafe urine-collection. Still, while undoubtedly important, this does not suffice to address the overall problem of exploitative emotional labor, for at least two reasons.

First, if exploitation of emotional labor is significantly pervasive in care contexts, the introduction of a new tool for addressing a specific need is probably insufficient for avoiding further versions of exploitation. Since the reasons why emotional labor is being exploited are typically not located in the internal dynamics of a specific care practice, but in the more general ideological and material system of care duties allocation, there is no guarantee that the practice would cease to be exploitative upon the introduction of the robot.

Second, and relatedly, because it leaves the wider structure in place, it can mask rather than downscale privileged irresponsibilities. The *wee-bot*, like other useful robots, can be an ally for nurses to perform their daily tasks better, but it can also be used by other stakeholders, notably employers and institutions, to elude their responsibilities for providing apt emotional labor conditions. Put differently, the *wee-bot* solution seeks to optimize demanding care conditions by accepting them as they are and finding more efficient ways for different care tasks to get done. In doing so, it fails to enable the critique of existing exploitative emotional labor conditions; in fact, it may mask those conditions by falsely assuring employers they have taken serious steps towards easing the work of their care workers. Such a movement would function as a new form of privileged irresponsibility by institutions and employers. Furthermore, since the impact of a new technology in a care practice is admittedly not fully predictable, the possibility for the *wee-bot* to create new emotional labor duties for care workers due to the need to smoothen and manage the emotional adaptation of patients to the new technology should be considered.

A second example, which looks at Paro, the much-studied seal-resembling companion pet robot, will make our concern more manifest. Paro is a social robot intended to convey the psychological benefits one can obtain from a pet while also adding some additional features of personal assistance thought to be convenient and useful in care contexts. It has been largely evaluated as a potential aid to people with dementia and their carers (Hung et al., 2019). Even though Paro is supposed to help reduce the workload of PCWs, the outcome of its introduction is not that straightforward. James Wright (2023) recently investigated the experience of a Japanese nursing home for people with dementia using a Paro robot. He found that, while residents seemed patently happy with the robot, the staff’s opinions were much more ambivalent. Among other implications, the robot increased their emotional labor: they were required to take care of newly emerging anxieties, fears, excessive attachments, jealousies, and other complex emotions aroused in the residents by the robot. Far from liberating the staff from some burdens, it created a new duty for constant supervision, which implied a high level of emotion management that was neither recognized nor supported.

This form of exploitative emotional labor can be understood as an already existing exploitative dynamics exerted by new means (robots), or as a new form of exploitation that may in turn require new tools for analysis. In our view, the better option for grasping the implications of robot-involving exploitative emotional labor is by attending to the social dynamics in place that can elicit exploitative practices by different means. That is, beyond robot design, which is undoubtedly relevant, the ways in which a specific society or group is incorporating robots in their care spaces and practices plays a role in how it impacts the potential exploitation of emotional labor.

Wright’s investigation helps us to take a step back in the analysis and look at the big picture that may be missing from CCVSD. He depicts the global image of the situation of assistive robotics in Japan, including the publicly-funded huge stake on them, the loneliness crises, the changes in family configuration, and the nationalistic ideology around care (Wright, 2023). The reliance on robots is motivated by the shortage of PCWs, and it runs parallel with new, sometimes controversial strategies of recruiting more PCWs. Caring institutions and public decision-makers are increasingly demanded to tackle the care crisis, and investing in robots can function as a form of responding to this demand without reorganizing care responsibilities in a fairer manner, as Tronto (2013) proposes.

The healthcare bet on robots, seen across the globe, can be claimed to serve as a new form of privileged irresponsibility: some actors, such as governments, mask their unaccountability by “contributing” to care in a different way. This different way may not actually be useful for a lot of care practices or PCWs, but serves as an excuse for continuing existing practices around the allocation of care responsibilities. At the same time, the emotional labor performed by some people is being exploited (they are the only ones deemed responsible for managing the challenging consequences of a new technological intervention that produces profit for others), and care values are not sufficiently acknowledged (they are more difficult to meet because of the workers’ overwhelming and institutions’ new way to excuse their unaccountability).

Tronto’s democratic allocation of care responsibilities would not mean that everyone is equally responsible for every care task. What she calls for is a public discussion on the reality of care needs, the fact that they are to be met somehow, and a democratic deliberation on the best way to distribute such duties. While this process, as any other democratic deliberation, does not guarantee that the model resultant will be the best sharing-model possible, it is aimed to start to unveil and tackle the current unfairness of care. Privileged irresponsibility stays in the way of a fair share, and they would be the first to be unmasked in such a democratic discussion. If robots serve as a way to continue exerting some forms of privileged irresponsibility, the care resulting from its introduction would still suffer from a tendency to exploit PCWs, which, in turn, prevents care values from flourishing fully. Even when PCWs manage to be attentive, responsible, competent, and responsive when performing their care duties within a context of roboticized care, and even when PCWs gain a sense of satisfaction from the care provided, care values cannot be said to be full if the implementation of a robot sustains old or enables new forms of privileged irresponsibility and exploitation. While CCVSD is undoubtedly a very valuable and useful resource at the level of design and at the level of care practices delivery’s assessment, it would benefit from being framed in a further political proposal regarding the structural conditions of care in contemporary societies. We argued that this is particularly important so as to safeguard against the employment of CCVSD in manners that mask rather than alleviate the exploitative dimensions of care’s emotional labor.

## Conclusions

Since some assistive robots are intended to be implemented in locations where care is performed professionally, it is relevant to consider how they can affect the labor conditions of PCWs. The category of emotional labor is a suitable tool for addressing the specificities of emotions in paid care work and for better understanding the effect robots may have on them. Emotional labor can be fulfilling, but also subjected to exploitation. Privileged irresponsibility, a concept proposed by Joan Tronto, helps to understand how emotional labor may be taken advantage of by some actors as a way to avoid a further reallocation of caring responsibilities in a fairer manner. Assistive robots may function as new tools for reinforcing this potentially exploitative trend and thus implicitly strengthen the vulnerability to exploitation of PCWs.

Van Wynsberghe’s CCVSD is a suitable tool for analyzing the sharing of responsibilities in each care practice, but it could benefit from new insights for acknowledging the conditions that shape how emotional labor is performed. Since systematic vulnerability to exploitation is not addressed in an exclusive design-based approach, its capacity to fully respect and foster care values is in question.

As mentioned in the introduction, our work can be understood as contributing to a more general endeavor to incorporate the value of “caring with” into the ethics of assistive robotics. Further research in this regard could include the potential unfairness regarding the quality of care people receive once robots are introduced in care systems at a broader societal scale. Given that the possibility of receiving good care is already unevenly distributed among groups, it is not unthinkable that robots could impact the distribution of quality care. Furthermore, since unfairness in care duties allocation is closely related to cultural attitudes towards care, further research should be also devoted to elucidating the effect robots may have on people’s perceptions of how care should be performed.

Specific consideration of the situation of PCWs is essential for a thorough ethical account of assistive robotics. In this paper, we have contributed to this general endeavor by addressing the specific problem of exploitation of emotional labor for PCWs. Importantly, our work shows how the impact of robots in any contexts is linked to social and political dynamics that need to be accounted for in the ethical analysis; inversely, that also means that the unfairness in care contexts cannot be solved by technological interventions alone, but only by structural social change. Ideally, the responsible development of assistive robotics would contribute to improving the conditions in which paid care work is conducted today. Whether and how this can be realized is an open question that warrants further research.

## Footnotes

1 The Care-Centred framework is designed for acknowledging care values in the design of robots, consisting of five aspects that should be evaluated: the context, the practice, the actors involved, the type of robot, and the manifestation of moral elements (Van Wynsberghe, 2015). Care-Centred Value-Sensitive Design is specific for the prospective design of new assistive robots, as a version of a Value Sensitive Design. Taken together, the CC and CCVSD frameworks offer a fine-grained lens for analyzing the implementation of assistive robots. In what follows, we will refer to the whole proposal as CCVSD.

2 We are grateful to an anonymous reviewer for encouraging us to emphasize this point here.

3 Assistive robots are also intended to function in households, where paid care work also exists. CCVSD can a priori be used in such contexts also. This possibility is further explained in Van Wynsberghe, (2016) but it is less developed than the healthcare version.

4 Whether the subjection of women can be understood as a form of exploitation has been discussed largely in Marxist feminist tradition (Dalla Costa & James 1975; Delphy, 1985), and some have even argued that the unfair allocation of care duties is a form of systematic exploitation of women by men (Bubeck, 1995).

5 Such recognition should not only unfold at the interpersonal level; it should also be operationalized in PCWs’ material-structural work conditions, e.g., in their work schedules, in the support and training offered to them, in shared spaces and opportunities provided for peer collaboration, and in compensations for the hard parts of the emotional work.
